# Clinical Evaluation of Corrosion Resistance, Ion Release, and Biocompatibility of CoCr Alloy for Metal-Ceramic Restorations Produced by CAD/CAM Technologies

**DOI:** 10.3390/dj11070166

**Published:** 2023-07-07

**Authors:** Zlatina Tomova, Angelina Vlahova, Stefan Zlatev, Ilyana Stoeva, Desislav Tomov, Delyana Davcheva, Viktor Hadzhigaev

**Affiliations:** 1 Department of Prosthetic Dental Medicine, Faculty of Dental Medicine, Medical University of Plovdiv, 3, Hristo Botev blvd, 4002 Plovdiv, Bulgaria; angelina.vlahova@mu-plovdiv.bg (A.V.); stefan.zlatev@mu-plovdiv.bg (S.Z.); viktor.hadzhigaev@mu-plovdiv.bg (V.H.); 2 Department of Diagnostic Imaging, Dental Allergology and Physiotherapy, Faculty of Dental Medicine, Medical University of Plovdiv, 3, Hristo Botev blvd, 4002 Plovdiv, Bulgaria; iliyana.stoeva@mu-plovdiv.bg; 3 Department of Bioorganic Chemistry, Faculty of Pharmacy, Medical University of Plovdiv, 15-A “Vasil Aprilov” blvd, 4002 Plovdiv, Bulgaria; desislav.tomov@mu-plovdiv.bg; 4 Department of Clinical Laboratory, Faculty of Pharmacy, University Multi-Profile Hospital for Active Treatment St. George Plovdiv, Research Institute at Medical University of Plovdiv, Medical University of Plovdiv, 15-A “Vasil Aprilov” blvd, 4002 Plovdiv, Bulgaria; delyana.davcheva@mu-plovdiv.bg

**Keywords:** dental alloys, corrosion, oxidative stress

## Abstract

Background. CAD/CAM technologies facilitate using powder CoCr alloys to produce metal-ceramic dental restorations. However, base alloys may induce oxidative stress in the oral cavity due to corrosion and ion release. This study evaluated resistance to corrosion and release of metal ions from 3D printed CoCr dental alloy and their effect on oral oxidative stress. Methods. Metal-ceramic crowns with 3D printed copings from CoCr alloy EOS CobaltChrome SP2 (EOS, Germany) were fabricated for 35 patients. Inductively coupled plasma mass spectrometry (ICP-MS) was used for measuring the concentration of Co and Cr ions in non-stimulated saliva before prosthetic treatment (BPT), at 2 h and 7 days after the dental treatment (APT2, APT7, respectively). Open circuit potentials (Eocp) were evaluated at APT2 and APT7. Estimating oral oxidative stress, measurements of 8-isoprostaglandin F2-alpha were conducted using liquid chromatography-tandem mass spectrometry (LC-MS/MS) at stages BPT, APT2, and APT7. Results. Salivary Co level increased at APT2 and decreased to the initial levels at APT7. No statistical difference was found between the levels of 8-isoPGF2-alpha measured, and between the Eocp measurements at APT2 and APT7. Conclusions. The studied alloy showed stable corrosion resistance and the metal ion release did not induce oral oxidative stress.

## 1. Introduction

With the rise in CAD/CAM technologies, new production methods and materials have been developed. Regarding digital innovations, CNC (computer numerical control) milling and 3D printing are the two main techniques applied in the dental field for production of prosthetic dental restorations. Dental alloys may be processed both by subtractive and additive methods [1]. After creation of the digital model and the CAD design, the STL-file is sent to the manufacturing module for production of the final restoration. Discs made of the required metal alloys are offered by the manufacturers for the milling technique. Because of the high hardness of the alloys which cause fast wear of the drilling instruments, discs of “green” presintered alloys are developed, which require postprocessing of the milled restoration. Some of the disadvantages of the subtractive methods are the waste of the material from the milling discs which cannot be used for another restoration and that the accuracy of restoration fabrication depends on the number of axes in the milling units [2]. For AM methods which include selective laser melting (SLM), selective laser sintering (SLS), direct metal laser sintering (DMLS), and electron beam melting (EBM), powder dental alloys are developed [3]. Mechanical properties, metal-ceramic bond strength, corrosion resistance, and structure depend on the alloy composition and the production method of the metal coping [4]. Nickel-Chromium, Cobalt-Chromium, and Titanium alloys are the three main types of base dental alloys most used in dentistry.

The tendency to corrosion of dental alloys is crucial regarding their biocompatibility. The deterioration of metal objects is caused by the action of different components of the surrounding medium. When a metal is placed in an electrolyte medium, a process of ionization is initiated on its surface and metal ions are released in the surrounding environment as the metal begins to dissolve. An oxide layer, which suppresses corrosion and limits it to the object’s surface, is formed by the interaction of the absorbed oxygen molecules and the metal ions [5,6].

All areas in the oral cavity are covered with a thin biofilm formed by proteins and glycoproteins from the saliva [7]. After fixing a prosthetic restoration into the patient’s mouth, this organic layer covers the construction and may also decrease the corrosion level. Despite the oxide passive layer formation and biofilm’s presence, the corrosion process of metal objects in the oral cavity may continue [8]. Moreover, electrochemical corrosion appears because of the electrolytes present—electric load on the metal surface can be measured. If metal objects are in direct contact or connect through a conductor (saliva, blood plasma, soft gingival tissues), a galvanic electric current between them will be formed. This process of oral electro-galvanism may affect the body in several ways—due to the electrical field developed, the increased corrosion damage of the metal surface, and the ion release [9,10]. Numerous factors may affect the corrosion rate of metal dental restorations. The presence of some microorganisms, the intake of soft drinks, food diet, and gastroesophageal reflux disease may change the acidity in the mouth, thus increasing the corrosion rates of the alloys [11,12].

The ions released from the metal dental restoration may affect not only the surrounding tissues but also distant areas and biological systems of the body [13,14]. They may also cause systemic and local toxicity, allergic reactions, and even carcinogenic alterations [15,16,17]. Cobalt is an important element for human health. It is a part of the molecule of vitamin B12, which is crucial for erythrocyte formation, DNA and fatty acids synthesis and regulation, and metabolism of amino acids. The daily necessary intake of Cobalt is around 2.4 μg, although the actual intake usually exceeds it and is around 5–40 μg. Gastrointestinal absorption is relatively low and depends on the solubility of the Cobalt compound which enters the body. If not used for Vitamin B12 formation, most of the absorbed Cobalt becomes attached to the serum albumin and other blood proteins and is eliminated through kidneys with urine [18]. Nevertheless, in high doses, Cobalt may cause acute toxic reactions or adverse effects in cases of continuous chronic exposition. It may induce hypoxic-like response and elevated levels of oxidative stress leading to a change in expression of genes and proteins in different cell lines [19]. Exposition to elevated Cobalt concentration may initiate cell apoptosis and decrease lymphocyte proliferation. Both Co2+ and Co6+ inhibit the release of Interleukin 2 and even if the metal ions do not possess direct cytotoxic effect, they may contribute to a shift in the immune system responses [17]. Cobalt ions are toxic to the lungs and are one of the respiratory tract’s major sensibilization agents. Studies show that excess intake of Cobalt ions has neurotoxic effects and may cause permanent loss of hearing ability [20,21]. The derived no-effect level (DNEL) of Cobalt taken via the oral route is 8.9 µg/kg bw/day [22]. Chromium as Cr 3+ takes part in glucose metabolism. It is present in nutrition supplies for patients with type II diabetes [23]. Studies show that Chromium is associated with numerous pathological processes including carcinogenicity [24]. Compared to Cr 3+, Cr 6+ has a better permeability through cell membranes and may cause intracellular reactive oxygen and nitrogen species (ROS, RNS) formation inducing an increase in oxidative stress. The change in the valency of the Chromium ion from 6+ to lower may affect DNA and cause mutations [25]. Prolonged exposition to low doses of Nickel and Chromium leads to their accumulation in kidneys and may cause renal dysfunction [14].

Numerous studies assessing metal ion release in laboratory conditions show the importance of the specimen fabrication method, the content of the alloy, the type of the medium, the acidity of the medium, and the period of stay [26,27,28]. However, these studies do not consider some biological factors, such as the content of saliva, oral bacteria, and mastication. There are a few studies evaluating ion release from dental alloys in clinical conditions. Garhammer et al. found a correlation between the metal objects in the oral cavity and the content of metal ions in saliva [29]. Siddharth et al. showed that the metal ion emission from dental prostheses is time-dependent and had the highest levels immediately after insertion in the oral cavity [30]. Singh et al. found the highest ion release in the first week after placement of the orthodontic devices [31]. According to other studies, increased ion emission is observed for several months after placing the restoration in the oral cavity [32].

Although nowadays CoCr dental alloys are one of the most preferred type of alloys for fixed metal-ceramic prosthetic restorations, according to the regulations of EU, their use must be restricted because of the potential adverse effect of Cobalt compounds [33].

Reactive oxygen species (ROS) are formed as normal products of human metabolism and take part in the signaling and defensive systems of the body. In a healthy organism, their formation and deactivation are in equilibrium. If this balance is disturbed, oxidative stress appears, leading to negative effects on biomolecules—lipids, proteins, and nucleus acids [34]. The effect of action of ROS on lipids may lead to the formation of potentially carcinogenic and mutagenic products and damage of the function and the structure of cell membranes. Cell death by apoptosis or autophagy may also be associated with the ROS [35]. The products of lipid peroxidation may be utilized as a marker for oxidative stress in the body [36]. Non-enzymatic peroxidation of arachidonic acid and its esters present in cell membranes is the main path of isoprostanes formation [37]. The low reactivity of isoprostanes and their presence in all body fluids make the group of isoprostanes an adequate candidate for oxidative stress biomarker. In addition, local isoprostane concentration may be used for evaluating and observing different body systems [38].

Oral oxidative stress level depends on several factors acting simultaneously or separately—inflammation in the oral cavity, smoking, food diet, and dental materials [39,40]. Numerous dental materials—composites, root canal obturating materials, alloys, glass ionomer cements, titanium implants etc., may lead to disturbance in the local formation and neutralization of ROS. It is proven that medical and dental biomaterials affect the prooxidant and antioxidant activity of the body because of their metal ion release [41]. Studies show that Co and Cr ion release from metal alloys may induce oxidative stress in specific cell cultures [19,25]. According to some authors, the cytopathological effect caused by free metal ions is due to induced oxidative stress changes [42]. However, according to the studies of Atuğ Özcan et al., orthodontic dental materials do not lead to oxidative damage in the oral structures [43].

The effect of corrosion and metal ion emission of base alloys on specific cell cultures is evaluated in laboratory conditions by numerous authors [17,44,45].

Our research aimed to assess the level and impact of corrosion resistance and ion release in saliva from CoCr alloy for DMLS in clinical conditions. The null hypotheses were that the metal ion emission due to the corrosion process is not time-dependent and would not increase local oxidative stress levels after cementing the metal-ceramic restoration in the oral cavity.

## 2. Materials and Methods

Informed consent was signed by all the participants included in the research. The procedures followed the standards of the Institutional Ethical Committee of the Medical University of Plovdiv, Bulgaria (Decision № С—03-2/10 April 2020) and the Association Declaration of Helsinki 1964. Patients were selected according to the following criteria: nonsmokers without allergies and acute or chronic diseases at the beginning of the study. Before the research, 85 patients with indications for prosthetic treatment were examined. Overall, 50 of them were excluded from the study—25 were smokers, 5 had diabetes, 8 had cardiovascular diseases treated with oral medication, 1 had chronic renal disease, and 11 needed fixed prosthetic restorations for partially edentulous alveolar ridges. No symptoms of gastric disorders were reported by the participants before the beginning of the treatment and no evidence of acidic lesions on hard dental tissues was detected.

**Figure 1:**
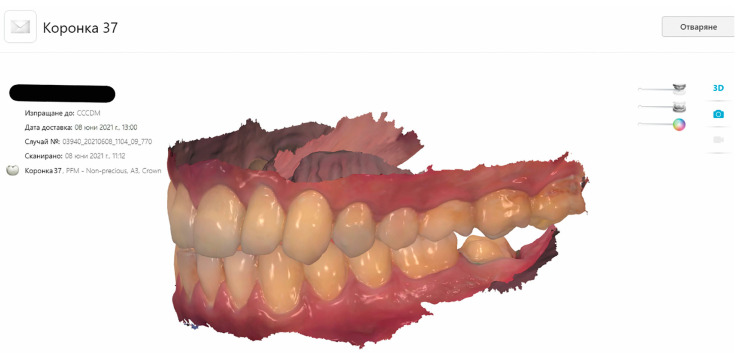
Digital impression after tooth preparation for a crown on the mandibular second molar. A screenshot from the Bulgarian version of 3Shape software.

Using CAD/CAM technology, metal ceramic crowns were produced for 35 patients. After intraoral scanning for creating digital impressions (Figure 1), the metal copings were designed and fabricated in the 3D printer EOS M100 (EOS, Krailling, Germany) for direct metal laser sintering (DMLS) at the CAD/CAM Center of the Research Institute, Medical University—Plovdiv. The parameters of work of the 3D printer were as follows: laser power—200 W; laser scanning velocity—7000 mm/s; layer thickness—20 μm.

The composition of the CoCr alloy EOS CobaltChrome SP2 (EOS, Krailling, Germany) used in the study according to the producer is presented in Table 1.

**Figure 2:**
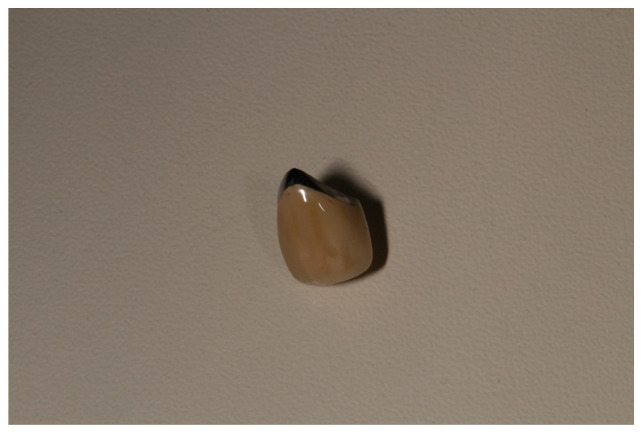
Exposed lingual metal edge of the metal-ceramic crown.

Three-dimensional printed resin models, fabricated in the Form 2 resin printer (Formlabs, Somerville, MA, USA), were used to complete the metal-ceramic restorations. The ceramic material VITA VM 13 (VITA Zahnfabrik H. Rauter GmbH & Co., Bad Säckingen, Germany) was applied for veneering the restorations. According to the design, an exposed metal area, 2 mm in height, was left lingually at the gingival margin (Figure 2). Resin-modified glass ionomer cement Ketac Cem Plus (3M ESPE, Saint Paul, MN, USA) was used for fixation of the crowns.

The dental visits were planned for the morning, at least two hours after breakfast or any hygienic procedures performed by the patients. According to the instructions, no food and drinks except water were taken by the patients before the dental appointments.

Samples of non-stimulated saliva were taken at three time-points: before any dental prosthetic treatment was performed (BPT), 2 h (APT2), and seven days post cementation (APT7). Samples were collected in the dental office by spitting in low-density polypropylene containers (Nuova APTACA, Asti, Italy). In term to avoid circadian rhythm variations, all patients were asked to come between 9.00 a.m. and 12.00 a.m. Furthermore, no stimulants—visual, taste, or aromatic, which could affect saliva flow, were present. During sample collection, patients were in seated position, with their heads slightly bent forward. The patient’s mouth was rinsed with distilled water, after which the saliva gathered at the bottom of the oral cavity was spat into the container. The procedure time was 15–20 min. From the collected 15 mL of non-stimulated saliva, 5 mL was placed in a centrifugal tube for measurement of isoprostane 8-isoPGF2-alpha, and the remaining 10 mL stayed in the initial container for elemental analysis. All labware used for saliva sampling, storage, and analysis was tested prior to the beginning of the study for contamination with Co and Cr. For this purpose, ten vessels of each package were filled with ultrapure water and 1% (*v*/*v*) HNO3 and kept for 24 h under ambient temperature. After the leaching step, the samples were analyzed by ICP-MS (Thermo Scientific, IcapQ, Waltham, MA, USA). All tested vessels were evaluated as appropriate for use, showing insignificant contamination levels with the selected trace elements for the analysis. All the samples were immediately frozen at −20 °C and later transferred for storage at −70 °C to the Research Institute at the Medical University of Plovdiv.

Cobalt and Chromium ion emission was assessed by ICP-MS (Inductively coupled plasma mass spectrometry) using Thermo Scientific iCAP Qc (Thermo Fisher Scientific, Waltham, MA, USA). The concentration of 8-isoPGF2-alpha was measured by LC-MS/MS (liquid chromatography with tandem mass spectrometry) with the system UHPLC Thermo Dionex Ultimate 3000 with mass detector Thermo Quantum Access Max (Thermo Fisher Scientific, Waltham, MA, USA). Before the study was performed, an LC-MS/MS method for the detection of 8-isoPGF2-alpha was developed and validated [46].

The clinical assessment of the resistance to corrosion of the studied alloy was conducted according to the open circuit potentials (Eocp) appearing on the visible metal surface of the crown using Dentotest Six (Atlantis, Plovdiv, Bulgaria) in “Pathogalvanism” mode without electrical current flow to or from the tested material. The calibration of the apparatus was conducted by voltage calibrator FLUKE SLK 753 and was according to the standard ISO 13485 (CE 2274). The active electrode of the apparatus was in contact with the lingual metal edge of the metal-ceramic crown and the reference stainless steel passive electrode was in contact with gingiva at a 2–4 mm distance. The Eocp values have been registered two hours and seven days after cementation of the prosthetic restorations, thus, to establish the change in the corrosion resistance in longer exposition in the oral cavity.

### Statistics

Statistical analyses were performed using G*Power v.3197 (Universität Kiel, Kiel, Germany) and R 4.05 (Lucent Technologies, Auckland, New Zealand Lucent Technologies, Auckland, New Zealand)) Sample size calculations were conducted with G*Power (Universität Kiel, Germany). The alpha level was set to 0.05 and statistical power to 0.8, which determined a minimum required sample size of 34 patients. Descriptive statistics and graphical analysis were used to characterize the studied variables. The Friedmann test was used to compare the ion expression and isoprostane levels, and a paired sample T-test to compare the Eocp values.

## 3. Results

**Figure 3:**
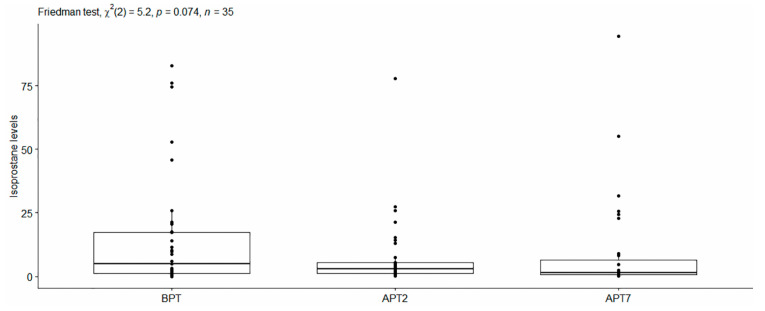
Levels of 8-isoPGF2-alpha in the studied time points.

Isoprostane levels. Descriptive statistics for the obtained values from the isoprostane levels at the different time points are presented in Table 2. The conducted Friedmann test did not reveal overall statistically significant differences in isoprostane levels for the studied time points [χ^2^(2) = 5.2, *p* > 0.05] (Figure 3).

ICP levels. Descriptive statistics for the obtained values from the ICP levels at the different time points for both studied ion expressions (Co and Cr) are presented in Table 3.

**Figure 4:**
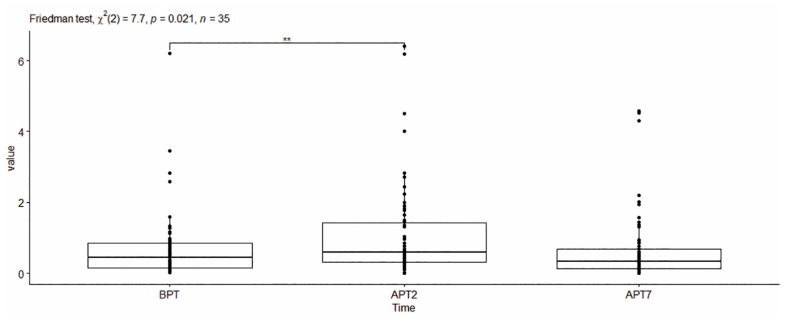
Values of Cobalt concentration in non-stimulated saliva. **—denotes statistically significant difference.

Cobalt concentration. The ICP values were statistically different for the different studied time points—χ^2^ (2) = 7.70, *p* < 0.05 with a small effect size [Wk = 0.11]. The pairwise Wilcoxon test revealed a significant difference in the expressed ICP levels for Co between the time points BPT-APT2 [*p* < 0.05] (Figure 4).

**Figure 5:**
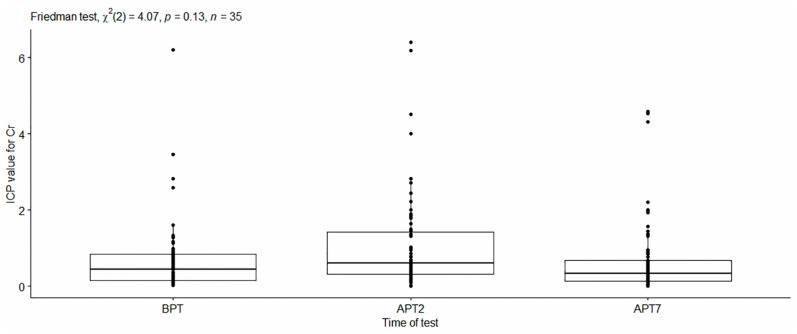
Values of Chromium concentration in non-stimulated saliva.

Chromium concentration. The ICP values were not statistically different for the studied time points—χ^2^ (2) = 4.07, *p* > 0.05. The dynamics of the Cr ion expression during the three studied time points are presented in Figure 5.

**Figure 6:**
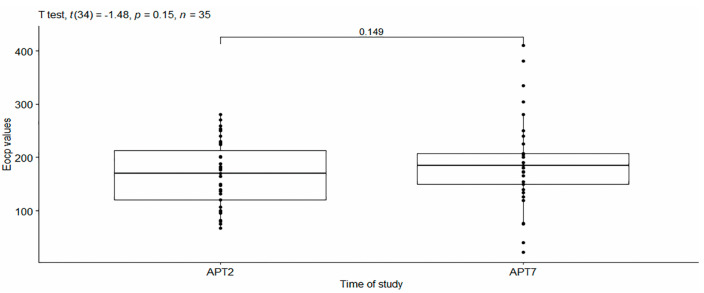
Open circuit potential values in the studied time points.

Eocp values. Descriptive statistics for the obtained values from the Eocp levels at the different time points are presented in Table 3. A pairwise *t*-test was used for assessment of the differences in the Eocp levels two hours and seven days after cementing the crown. The results did not show a statistically significant difference in the two studied time points [t (34) = 1.48, *p* > 0.05] (Figure 6).

## 4. Discussion

The research hypothesis that metal ion emission is not time-dependent was rejected, but the hypothesis that detected metal ion emission would not increase the local oxidative stress level was accepted.

Co ion release is evident on the 2nd hour after placement of the metal-ceramic crown in the mouth. According to the available online safety data sheet for EOS SP2 from 2018, the DNEL for Cobalt is 29.8 µg/kg bw/day [47]. However, recently the DNEL for Cobalt received via oral route has been lowered to 8.9 µg/kg bw/day [22]. The highest mean values of Co ion release found at 2nd-hour measurement (stage APT2) in the study are below the revised DNEL, and no carcinogenic effect is expected.

On the 7th day, the values of Co concentration in saliva decreased to the initial levels. This finding corresponds with the studies of Siddharth et al., who found the maximal ion release from the alloy immediately after placement in the oral cavity [30]. According to our results, the ion release from the metal alloy on the 7th day is negligibly low and would not affect the metal ion content in saliva. Our results do not confirm the findings of Velasco-Ibáñez et al. [32], who found metal ions in saliva from the dental restorations up to six months after placement. The different type of alloy chosen for assessment may be the reason for the different outcome of our research compared to the abovementioned author.

The composition of the alloy and the production method are of crucial significance for the properties of the final restoration and corrosion behavior in clinical conditions. According to Padros et al., 2020 the method of producing the dental restoration—traditional casting, CAD/CAM milling, or laser sintering, plays an important role regarding properties of the alloy because of the difference in the received final internal structure and surface composition [48].

The insignificant change in Chromium concentration in our research disagrees with some reported findings. According to Barrett et al., the release of Chromium ions increases in the first two weeks and decreases in the following two weeks [49]. Studies by Amini et al. on the salivary concentration of Chromium showed a slight increase after the placement of orthodontic devices. A twelve-month period is needed to reach the initial Chromium levels [50]. Singh et al. concluded that the maximal concentration of leaching Chromium ions was observed seven days after the dental metal device was placed in the oral cavity [31]. The lack of increase in salivary Chromium concentration after placement of the metal-ceramic crowns may be explained with the small area of exposed metal crown edge and the rapid formation of a stable surface layer of Chromium oxide [8].

In the conducted in vivo study, the saliva samples from all the participants were collected between 9.00 a.m. and 12.00 a.m. and at least two hours after taking any food, to make the salivary flow comparable and to eliminate the effect of diet on the ion emission and corrosion rates. However, after consumption of food or soft drinks, the acidity in the oral cavity increases and a risk of increased ion emission, which cannot be detected in the study, appears [12]. Furthermore, the possible intake of some metal ions from microorganisms present in the dental plaque must be taken into consideration [51].

The interaction of cell membrane lipids with ROS leads to formation of 8-isoPGF2-alpha. Isoprostane concentration represents an indicator for possible free radical damage of human cells. Some dental materials may induce oral oxidative stress because of inflammatory processes appearing in the mucogingival and periodontal tissues [38,39]. Our results showed no significant change of free 8-isoPGF2-alpha in the samples of non-stimulated saliva 2 h and 7 days after the completed prosthetic treatment. The change in Cobalt ion release from the metal-ceramic alloy did not affect the concentration of the tested isoprostane in non-stimulated saliva. Our results do not correlate with the conclusions of some authors, according to which CoCr alloys cause elevation in the level of oxidative stress [52]. Our research confirms the findings of other scientists which showed that dental CoCr alloys do not cause pathologic oxidative stress changes [53]. The hypothesis that elimination or decrease in inflammation level may reduce the oxidative stress rate is supported by the studies of some researchers [54,55]. According to their results, improved measures of oral hygiene remove some of the ROS-producing bacteria together with the dental plaque and increase the level of crevicular fluid delivering antioxidant agents. The constant rate of salivary 8-isoPGF2-alpha found in the patients included in our study at stages APT2 and APT7 may be due to the action of two independent factors–the improved oral hygiene measures during dental treatment, which may have led to a decrease in soft tissues inflammation rate from one side, and the potential increase in ROS formation induced by the metal release from the other.

Open circuit potential measurements showed no significant difference between the values received on the second hour (APT2) and the seventh day (APT7) after placement of the metal-ceramic crowns. From the results, it may be assumed that a 2 h period is enough for surface passivation, after which high and stable corrosion resistance occurs [4]. Besides the surface oxide layer, formation of a biofilm may also affect the resistance to corrosion of the alloys. According to Lu et al. S. mutans in oral biofilms may improve the corrosion resistance of dental alloys by creating a physical barrier that intercepts oxygen interaction with the surfaces of metal objects [11]. The constant Eocp values suggest that no oral electro galvanic effects are expected in the studied group of patients [9].

The small size of the visible metal surface of the produced metal-ceramic crowns may be assumed as a limitation of our study. It may be assumed that if a bigger area of the metal alloy is exposed to the influence of the orally acting factors, the ion release may increase and a change in the oxidative stress level might be detected. A potentially harmful Cobalt emission may appear within the first week after placement in cases when larger dental restorations are needed.

Our findings have significant clinical implications. The studied CoCr alloy for metal-ceramic restorations produced by CAD/CAM technologies is a biocompatible material that may be successfully used by dental practitioners and technicians, knowing that the alloy possesses stable corrosion resistance and minimal ion release which do not elicit oxidative stress, which contributes to the longevity and safety of dental restorations, ensuring the well-being of patients.

## 5. Conclusions

Based on the results of the study and its limitations, the following conclusions can be outlined:

1. Co ion emission significantly increases 2 h after intraoral placement.
2. Seven days after placement in the oral cavity, the metal ion release from the studied alloy is insignificant compared to the initial metal ion concentration in non-stimulated saliva.
3. No correlation exists between the metal ion emission and the concentration of 8-isoPGF2-alpha in non-stimulated saliva.
4. Regarding the constant in-time Eocp values, the studied dental alloy has high and stable resistance to corrosion.

## Figures and Tables

**Table 1:** Elemental composition of dental alloy EOS SP2 (EOS, Germany).

Element	Wt-%
Co	63.8
Cr	24.7
Mo	5.1
W	5.4
Si	1
Fe	Max. 0.5
Mn	Max. 0.1
Free of Ni, Be, Cd, and Pb according to ISO 22674	

**Table 2:** Descriptive statistics of 8-isoPGF2-alpha in the different time points (ng/L).

	Variable	n	Min	Max	Median	Mean	sd
BPT	value	35	0	82.79	4.84	15.13	23.022
APT2	value	35	0.11	77.93	2.85	7.543	14.171
APT7	value	35	0.04	94.54	1.48	8.959	18.984

**Table 3:** Descriptive statistics of Co and Cr concentrations in the different time points (µg/L).

Ion	Time	Variable	n	Min	Max	Median	Mean	sd
Co	BPT	value	35	0.02	3.45	0.22	0.581	0.831
	APT2	value	35	0.01	6.18	0.56	0.992 *	1.276
	APT7	value	35	0.01	4.58	0.18	0.628	1.232
Cr	BPT	value	35	0.02	6.21	0.66	0.777	1.025
	APT2	value	35	0.19	6.41	0.64	1.138	1.27
	APT7	value	35	0.08	2.21	0.47	0.697	0.605

* Level of significance between BPT and APT2 *p* = 0.021 (Wilcoxon test).

## Data Availability

Data can be provided by the corresponding author upon request.
